# Epigenetic Mechanisms Modulated by Glucocorticoids With a Focus on Cushing Syndrome

**DOI:** 10.1210/clinem/dgae151

**Published:** 2024-03-22

**Authors:** Ticiana Paes, Richard A Feelders, Leo J Hofland

**Affiliations:** Department of Internal Medicine, Division of Endocrinology, Erasmus Medical Center, 3015 GD Rotterdam, The Netherlands; Division of Endocrinology, Diabetes and Hypertension, Brigham and Women's Hospital and Harvard Medical School, Boston 02115, MA, USA; Department of Internal Medicine, Division of Endocrinology, Erasmus Medical Center, 3015 GD Rotterdam, The Netherlands; Department of Internal Medicine, Division of Endocrinology, Erasmus Medical Center, 3015 GD Rotterdam, The Netherlands

**Keywords:** epigenetic, glucocorticoids, Cushing syndrome

## Abstract

In Cushing syndrome (CS), prolonged exposure to high cortisol levels results in a wide range of devastating effects causing multisystem morbidity. Despite the efficacy of treatment leading to disease remission and clinical improvement, hypercortisolism-induced complications may persist. Since glucocorticoids use the epigenetic machinery as a mechanism of action to modulate gene expression, the persistence of some comorbidities may be mediated by hypercortisolism-induced long-lasting epigenetic changes. Additionally, glucocorticoids influence microRNA expression, which is an important epigenetic regulator as it modulates gene expression without changing the DNA sequence. Evidence suggests that chronically elevated glucocorticoid levels may induce aberrant microRNA expression which may impact several cellular processes resulting in cardiometabolic disorders.

The present article reviews the evidence on epigenetic changes induced by (long-term) glucocorticoid exposure. Key aspects of some glucocorticoid-target genes and their implications in the context of CS are described. Lastly, the effects of epigenetic drugs influencing glucocorticoid effects are discussed for their ability to be potentially used as adjunctive therapy in CS.

In Cushing syndrome (CS), adrenocorticotropic hormone (ACTH) hypersecretion by a pituitary adenoma or an ectopic source, or autonomous cortisol hypersecretion by an adrenal tumor, induces chronic endogenous hypercortisolism with loss of the cortisol circadian rhythm (1). CS is more prevalent in women than men and frequently occurs in the fourth to sixth decades of life (2).

Glucocorticoids (GC) have extensive physiological actions and regulate up to 20% of the expressed genome, mainly related to the immune system, metabolic homeostasis, and cognition. Therefore, the prolonged exposure to high cortisol levels results in a wide range of devastating effects, including major changes in body composition (obesity, muscle atrophy, osteoporosis), neuropsychiatric disturbances (impaired cognition, depression, sleep disturbances), the metabolic syndrome (obesity, hypertension, insulin resistance, and dyslipidemia), hypercoagulability, and immune suppression (3, 4). The consequences of hypercortisolism lead to compromised quality of life and increased mortality rate (5). The mortality rate in patients with CS is 4 times higher than the healthy control population (6). Risk factors such as obesity, diabetes, and hypertension contribute to the increased risk of myocardial infarction, stroke, and cardiac insufficiency. As a result, cardiovascular disease is the leading cause of the premature death in CS (5). Infectious disease is also an important cause of death in CS (5). Therefore, prompt treatment to control hypercortisolism is imperative to prevent complications and an increased mortality rate.

Despite the efficacy of treatment leading to disease remission, the clinical burden of CS improves, but does not completely revert, in every patient (7). Indeed, obesity, neuropsychiatric disturbances, hypertension, diabetes, and osteoporosis persist in a substantial number of biochemically cured patients. For instance, in a study involving 118 CS patients in remission for about 7.8 years (median), resolution of comorbidities such as diabetes occurred in only 36% of cases, hypertension in 23% of cases, and depression in 52% of the cases (8). It has been proposed that epigenetic changes as a consequence of hypercortisolism is a mechanism of the persistence of some comorbidities (9-12).

Epigenetics is a reversible process that modifies gene expression without any alterations in DNA sequence; frequently it is mediated by histone modification and DNA methylation together with microRNAs (13-15). GCs use the epigenetic machinery as a mechanism of action to regulate gene expression in physiological circumstances, such as metabolic actions and stress response. Its networks involve DNA and histone modifying enzymes, such as DNA methyltransferases (DNMTs), histone acetyltransferases (HATs), and histone deacetylases (HDACs) (16). (Fig. 1) The DNA methylation process catalyzed by DNMTs is usually associated with downregulation of gene expression (17). Histone modifications catalyzed by HAT enzymes induce gene transcription, while those by HDAC enzymes induce transcriptional repression (17). Drugs interfering with these enzymes (so-called epigenetic drugs) may affect the GC genomic actions confirming the interaction between GC and the epigenetic system (18, 19). Furthermore, GC can modulate HDAC and DNMT expression and activity (16, 19, 20). Based on these data it might be speculated that in CS, epigenetic modifications induced by long-term GC exposure plays a role in the development of the disease-specific morbidity (9, 10).

**Figure 1.**
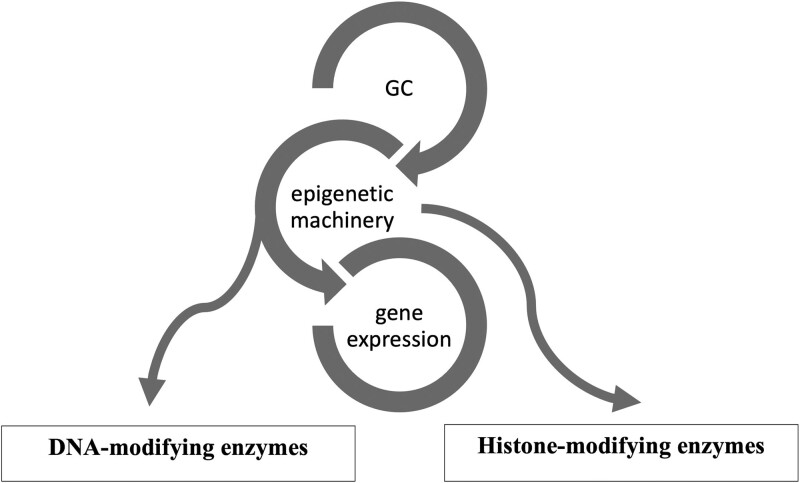
Glucocorticoid (GC) and its epigenetic machinery. GC through its receptor interacts with DNA and histone modifying enzymes, such as DNA methyltransferases (DNMTs), histone acetyl transferases (HATs), and histone deacetylases (HDAC) to modulate gene expression.

In this review we provide an overview of epigenetic aspects of GC action in physiological conditions and in the context of CS. We start with a detailed characterization of how GC, using the epigenetic system, can change chromatin structure in order to activate or silence gene expression. (Fig. 2) Subsequently, we describe the role of epigenetic mechanisms in the regulation of expression of several GC-target genes related to CS. Finally, we present the current evidence of epigenetic changes caused by the long-term of GC exposure and the potential use of epidrugs influencing GC actions.

**Figure 2.**
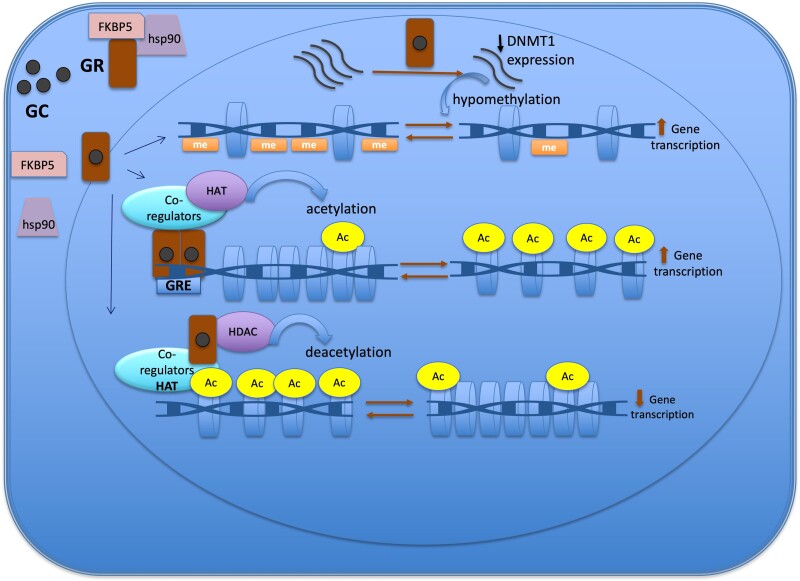
Epigenetic mechanisms of the glucocorticoid action to regulate gene expression. The GR is located in cytoplasm in a multi-protein complex; after GC binding, GR dissociates from the multi-protein complex, crosses the nuclear membrane, dimerizes, and binds to the GRE of the target gene. One of the mechanisms of action of GC is through the recruitment of co-regulators together with epigenetic enzymes, such as HAT, to change the chromatin structure, resulting in activation of gene transcription. Also, GR decreases gene expression by tethering other transcriptional factors and recruiting HDAC2, causing histone deacetylation, which leads to a repressed chromatin. GC can cause hypomethylation through downregulation in the expression of DNMT1. Abbreviations: Ac, acetylation; DNMT1, DNA methyltransferase 1; GC, glucocorticoid; GR, glucocorticoid receptor; GRE, glucocorticoid responsive elements; HAT, histone acetyltransferase; HDAC, histone deacetylases; Me: methylation.

## Search Strategy

A search of the PubMed database was conducted using the advanced search builder tool for articles in the English language on the following terms “*glucocorticoids*,” “*glucocorticoid receptor*,” “*Cushing*,” “*hypercortisolism*,” “*epigenetic*,” “*DNA methylation*,” “*histone deacetylase*,” “*histone acetyltransferase*,” “*microRNA*” “*fkbp5*,” “*clock genes*,” and “*POMC*.” Moreover, references were identified directly from the articles included in this manuscript. The articles were selected by the authors after being carefully analyzed regarding their importance and impact.

## Epigenetic Aspects of Genomic Action of Glucocorticoids

GCs regulate gene expression positively or negatively. GC-responsive genes include genes encoding for proteins associated with inflammation, metabolic processes, blood pressure and fluid homeostasis, apoptosis, cell cycle progression, circadian rhythm, and intracellular signaling (21).

The GC actions are cell type–specific (22). For instance, in an in vitro study, the comparison of GC-expressed genes between 2 cell lines, corticotroph (AtT20) and mammary (3134) cell lines, showed a different set of GC-regulated genes, revealing the cell type–specific nature of GC effects (23). GC function depends on the accessibility of glucocorticoid receptor (GR)-binding sites in the DNA of the target tissue, which in turn is mostly established during cell differentiation. Therefore, different chromatin organization explains the distinct GR-binding sites among different tissues (22, 24, 25). The chromatin accessibility is determined by histone modifications such as acetylation, methylation, phosphorylation, and/or DNA methylation, processes that are both dynamic and reversible (26).

Furthermore, gene expression is regulated in a GC-concentration-dependent manner which is tissue-specific. Only a few genes can be upregulated or downregulated at low concentrations of GC. For example, a dose of dexamethasone (Dex) as low as 0.5 nM selectively activated *PER1* (period 1, transcription factor related to circadian rhythm) expression in lung cancer (A549) cells (21, 27). Additionally, continuous GC exposure or pulsed GC (cortisol fluctuation during circadian rhythm) may cause different responses with respect to gene expression (26, 28). For example, constant treatment with corticosterone induced higher levels of *PER1* clock gene mRNA expression compared with pulsatile treatment, as demonstrated in an in vitro study using 3134 cell line (28).

The time course for gene expression in response to Dex is fast, with repression occurring slightly slower compared to activation. Half of activated and repressed genes are detected within, respectively, about 40 minutes and 53 minutes following Dex exposure (21).

In short, the transcriptional output in response to GC depends on cell type, as well as on the duration and intensity of GC exposure (21, 24, 26, 27). GCs act as a transcriptional regulatory factor resulting in activating or repressing the expression of genes. The GC exerts its function through binding to corticosteroid receptors, specifically, the mineralocorticoid receptor and the GR, members of the nuclear receptor superfamily (29, 30).

### Glucocorticoid Receptor

The GR is located in the cytoplasm in a chaperone complex which includes heat-shock proteins (70 and 90) and immunophilins (such as FK506 binding protein [FKBP5]). Cortisol diffuses across the cell membrane and binds with high affinity to the GR. The activated GR bound to GC dissociates of the multi-protein complex and is transferred to the nucleus, where it ultimately regulates gene expression (26, 31).

GR is a transcription factor encoded by nuclear receptor subfamily 3, group C member 1 (NR3C1) gene, located in chromosome 5, and consisting of 9 exons. It is composed of 3 major functional domains, namely a DNA binding domain (DBD), the C-terminal ligand-binding domain (LBD) and the N-terminal domain (NTB). The LBD recognizes and joins the GC. NTB contains an activation function-1 (AF1) which connects with co-regulators and the members of the general transcription machinery to activate target genes. The DBD comprises 2 zinc fingers motifs that are able to identify and bind to glucocorticoid responsive elements (GREs) (32, 33).

GRα is the most expressed and functionally active GR. GRβ is another isoform which is the result of an alternative splicing in exon 9 of the GR transcript. The difference between the 2 isoforms is the distinct ligand-binding domain in GRβ. This variance prevents the GRβ from binding to GC. In fact, the GRβ counteracts GRα function by interfering with its binding to a GRE in the target gene, and GRβ expression is associated with GC resistance (32). In addition, GRβ has its own transcriptional activity which is independent and distinct from GRα (34).

Another splice variant of human GR, GRγ, is associated with GC resistance in lung cell carcinoma and childhood acute lymphoblastic leukemia (33, 35). There is an additional amino acid (arginine) in the DBD of the GRγ that reduces, by about half, the capacity to activate or suppress the transcription of the target gene, as compared with GRα (32). One study identified GRγ in a small series of corticotroph adenomas (36).

### Glucocorticoid Mechanism of Action

The GR-GC complex induces or represses gene expression directly by binding to DNA, indirectly by tethering other transcription factors or yet in a composite manner that consists in binding DNA in association with binding to other co-regulators (35, 37).

The GR has the ability to reorganize the chromatin structure to become more or less accessible to the transcriptional machinery. In the classical mechanism of direct induction of gene expression, the GR dimerizes and binds to a GRE in DNA. The receptor recruits co-regulators, such as CREB binding protein, which has intrinsic histone acetyltransferase (HAT) activity that modifies the chromatin structure from an inactive to an active state. This model, called transactivation, upregulates the expression of some genes related to glucose, protein, and fat metabolism. Gene repression, on the other hand, is accomplished by GR binding to a negative GRE (nGRE) leading to the formation of a chromatin remodeling complex composed by co-repressor factors, such as NCOR1 and SMRT, and histone deacetylases (HDACs), that ultimately turn chromatin less accessible and suppress gene transcription. The gene repression through direct binding events occurs less frequently when compared to gene induction (25, 35, 38).

Another mechanism of GC action is through binding to other transcription factors (tethering). In case of switching off inflammatory genes, GR binds to transcriptional co-activator molecules, such as CREB binding protein with intrinsic HAT activity, and subsequently recruits HDAC2 to reverse histone acetylation, thus resulting in a suppression of the activated inflammatory gene (39). In the same model, GC interacts with other cofactors, such as the STAT family, to induce chromatin modifications resulting in increased gene expression (26).

Furthermore, the transcriptional dynamics of some genes follow a composite manner. In this model, GR, in conjunction with binding to GRE, also interacts with cofactors in order to enhance or reduce gene expression (35).

GCs can also modulate gene expression by influencing the transcription of epigenetic modifiers. An experimental study demonstrated that GC mediated the upregulation of HDAC2 in rats exposed to chronic stress, which in turn decreased the transcription of histone methyltransferase (Ehmt2) that ultimately upregulated the expression of Nedd4. Nedd4 is a ubiquitin ligase, expression of which has been related to cognitive impairment (40). Additionally, GC was found to interact with another epigenetic eraser, namely JMJD3, a histone demethylase, suppressing its transcription in endothelial cells treated with TNFα that led to decreased expression of other genes related to the blood-brain barrier (41).

GCs have the ability to induce (de)methylation changes in DNA, ultimately affecting gene expression. The DNA methylation process triggered by GC involves the family of DNA methyltransferases (DNMT) and ten-eleven translocation (TET) protein (20, 42-44). The DNMT, DNMT1, DNMT3A, and DNMT3B are able to transfer a methyl group to a cytosine residue in DNA, forming 5-methylcytosine (5mC), which negatively impacts gene expression. In contrast, TET protein chemically modifies the 5mC to form 5-hydroxymethylcytosine (5hmC), which ultimately leads to unmethylated cytosine, positively influencing gene expression (45).

Glucocorticoids mainly induce loss of methylation events rather than gain of methylation across the genome (11, 46). The DNA demethylation process can be either active or passive. The active mechanism is linked to the upregulation of TET enzyme expression that follows GC treatment, which was described in retinal and osteocyte cell line model studies (42, 43). The passive demethylation event involves the downregulation (Fig. 2) or dysfunction of DNMT1. DNMT1 is responsible for maintaining the methylation process in dividing cells (45). In case of GC exposure, GC can cause hypomethylation through downregulation in the expression of DNMT1, a process described in the AtT20 corticotroph tumor cell model, or through GC hindering DNMT activity, particularly DNMT1, as demonstrated in the retinal cell (RPE) line (20, 42, 44).

### Glucocorticoid-Induced Epigenetic Changes

There are several molecular mechanisms connecting GR activation and epigenetic modifications ultimately affecting gene expression (Fig. 2). As described above, GC uses epigenetic machinery, such as DNA and histone modifying enzymes, to restructure the chromatin in order to induce or silence gene transcription (16, 47).

In an in vitro study using murine AtT20 corticotroph tumor and neuronal cell lines, after chronic GC exposure followed by a recovery period in the absence of GC, the cells retained an “epigenetic memory” with persistence of loss of methylation content in *FKBP5* gene but with no increased gene expression at baseline. The functionality of this “epigenetic memory” only became evident in a second exposure to GC, when the cells responded sharply with a more robust expression of *FKBP5* gene compared to the cells without previous exposure to GC (44). Another in vitro study, using a human fetal hippocampal cell line, confirmed long-lasting DNA methylation changes induced by GC. The cells were treated for 10 days with dexamethasone, during the proliferative and cell differentiation phases of the cell line, followed by 20 days without any treatment. The second exposure to GC resulted in an enhanced gene expression of a subset of GC-target genes (48). Additionally, using an animal model subjected to chronic stress, a distinct gene expression profile was demonstrated in response to acute GC challenge compared to those without chronic stress history. The proposed mechanism was that chronic stress resulted in GC-induced enduring epigenetic changes in target genes, altering the responsiveness to a subsequent GC exposure (49).

In general, it seems that the majority of differential methylation regions (DMRs) induced by GC are loss of methylation rather than gain of methylation. In an experimental study, an association between hypomethylation and GC exposure was demonstrated in mice previously exposed to high levels of GC. Further analysis demonstrated that the genes linked with DMR were mostly related to metabolism, the immune system, and neurodevelopment (11).

Human studies have also shown that excess of cortisol can induce modifications in DNA methylation. DNA methylation data obtained from whole blood samples from patients with chronic obstructive pulmonary disease (COPD) treated with GC revealed DMR at specific CpG dinucleotides across the genome. These DMR were confirmed by pyrosequencing and annotated to genes, such as *SCNN1A*, encoding the α subunit of the epithelial sodium channel, *GPR97*, encoding G protein coupled receptor 97, and *LRP3*, encoding low-density lipoprotein receptor-related protein 3 (50). Furthermore, it has been proposed that the negative impact of chronic GC exposure on the immune system, which increases the risk of opportunistically infections, may be epigenetically mediated (51). In a clinical study, using whole blood samples, an analysis of genome-wide DNA methylation was performed on patients before and after exposure to GC (51). Long-term GC exposure disrupts, through a persistent modification of the cytosine methylation pattern, the mTORC1 pathway which affects CD4+ T cell biology (51).

Taken together, these data clearly show the interplay between GC signaling and methylation and histone modifications processes suggesting that GC interferes in the epigenetic landscape modulating gene expression. It is possible that most of these GC-induced epigenetic events are dynamic and temporary, while others may persist leading to long-lasting disorders. Further research to provide insight into what makes some events reversible is warranted.

## Epigenetic Changes as a Consequence of Long-Term Glucocorticoid Exposure in Cushing Syndrome

The comorbidities associated with CS are associated with increased mortality mainly due to cardiovascular events (52). GC-induced comorbidities in CS may be at least in part epigenetically mediated. Previous study using whole blood methylation profile demonstrated that specific hypomethylated CpG sites induced by GC were associated with Cushing comorbidities, such as hypertension and osteoporosis (46). The study identified a methylator predictor of GC excess which could be used as a biomarker to monitor GC status (46).

The long-term exposure to high cortisol levels may be crucial for the persistence of some morbidities in CS through epigenetic changes. Hypercortisolism-induced persistent changes in visceral adipose tissue gene expression through epigenetic modifications was investigated in a translational study (12). This study combined data from patients with active CS and data from an animal model of CS in active and remitted phase. Interestingly, the study demonstrated long-lasting changes in the transcriptome of adipose tissue that were associated with histone modifications induced by GC. Therefore, these epigenetic fingerprints observed even after the resolution of hypercortisolism may elucidate the mechanism of persistent modifications in gene expression in the visceral adipose tissue (12).

With regard to the persistence of GC-induced DMR, a genome-wide DNA methylation analysis showed a lower average of DNA methylation in patients in remission of CS compared to controls. Interestingly, the most common biologically relevant affected genes were retinoic acid receptors, thyroid hormone receptors, or hormone/nuclear receptors, important genes related to intracellular pathways and regulators of gene expression (9).

In summary, this large body of evidence supports the concept that prolonged GC exposure modulates the epigenetic landscape across the genome by inducing DMR and histone modifications. Some epigenetic modifications are persistent, and this may partially explain the incomplete reversibility of some of CS features following clinical remission.

## Glucocorticoid-Target Genes in Cushing Syndrome

A detailed identification and characterization of GC-target genes may shed light in the understanding of the pathophysiology and treatment response in patients with CS. For instance, the GC regulation of pro-opiomelanocortin (POMC) expression as part of the physiologic GC negative feedback may be impaired in Cushing disease (CD), which is an important mechanism for the maintenance of high GC levels (53). Another example is the interaction between GC and clock genes, which may interfere in the loss of the GC circadian rhythm and may contribute to metabolic disorders in CS (54). Furthermore, the suppressive action of GC on drug targets, such as the somatostatin receptor (subtype 2), may influence the efficacy of first-generation somatostatin receptor ligands in normalizing cortisol levels in CD (55). Here we describe how GCs using epigenetic machinery influence the expression of important target genes and their implications in CS.

### FKBP5

FK506 binding protein (FKBP5) plays an important role in the regulation of hypothalamic-pituitary-adrenal (HPA) system (56). As part of the GC negative feedback loop, GC binds to hypothalamic and pituitary GR. In the cytoplasm, GR is bound to a multi-protein complex including FKBP5. FKBP5 modulates GR action by decreasing GR binding affinity to GC and by preventing GR translocation from cytoplasm to nucleus (57, 58). In other words, an increase of FKBP5 expression is inversely correlated with GR activity and results in GC resistance leading to an impaired negative feedback regulation in the HPA axis (59).

*FKBP5* is a GC-responsive gene; its upregulation by GC is part of an intracellular negative short-feedback loop (60). The mechanism by which GC regulates *FKBP5* expression was shown to include inhibition of DNA methylation (44). In a model for CS, mice treated with corticosterone for 4 weeks had a reduced level of DNA methylation of *FKBP5* in DNA extracted from whole blood, which was strongly correlated in a negative manner with GC concentration. Interestingly, a negative correlation was also observed between the degree of *FKBP5* gene methylation measured at 4 weeks of GC exposure and the percentage of mice visceral fat (61). Accordingly, previous studies have provided compelling evidence of decreased methylation in the *FKBP5* gene in patients with active CS compared to healthy control (10, 46). Even in patients with CS in remission, previous data have suggested a small decrease in *FKBP5* methylation levels compared to healthy controls (9, 10). In an in vitro study, it was demonstrated that, by decreasing DNMT1 expression, GC is able to reduce FKBP5 methylation levels and, therefore, increase its expression (44).

Likewise, *FKBP5* mRNA is also sensitive to GC exposure. A time-dependent increase in blood FKBP5 mRNA after single-dose prednisone administration has been demonstrated in healthy humans (62). Accordingly, patients with ACTH-dependent CS had higher blood *FKBP5* mRNA levels compared with healthy controls, and after a successful surgery, *FKBP5* mRNA returned to baseline levels (63). Furthermore, in another study, blood *FKBP5* mRNA was inversely correlated with *FKBP5* promoter methylation and positively correlated with 24-hour urine free cortisol (UFC) levels in patients with CS (46). Taken together, this fine-tuning of *FKBP5* DNA methylation and mRNA according to the level of GC suggests that *FKBP5* can be used as a biomarker to infer the magnitude of GC exposure.

### *POMC* and Corticotropin-Releasing Hormone

The partial resistance of the corticotroph adenoma to GC negative feedback is a hallmark of CD. Indeed, the lack of this inhibitory effect constitutes a method to diagnose CD, that is, with the dexamethasone suppression test. One of the mechanisms related to the insensitivity to GC can be attributed to GR mutations which are, however, rarely found in corticotrophinomas (64). Another mechanism that was uncovered in corticotroph adenomas is an overexpression of the HSP90 chaperone resulting in reduced affinity of GR to its ligand and consequently GR resistance (53, 65).

In addition, the loss of protein expression of either Brg1, ATPase component of the SWI/SNF chromatin remodeling complex, or HDAC2 has been linked to GC resistance in about 50% of some adenomas (66). The trans-repression process on POMC transcription achieved by GC involves both the histone deacetylation enzyme and Brg1. One mechanism of corticotropin-releasing hormone (CRH)-induced POMC expression is through an orphan nuclear receptor (NR) related to NGFI-B (Nur77). NGFI-B binds to the NurRE sequence in the promoter region of POMC gene and recruits a co-activator to mediate its transcription. In a tethering mechanism, the GR directly interacts with NGFI-B to form a *trans*-repression complex, which contains the GR itself, Brg1, the nuclear receptor, and HDAC2; the latter being essential to block the gene expression through chromatin remodeling process (53, 66).

In CD, hypercortisolism exerts a negative feedback at CRH secretion from the hypothalamus (67). The mechanism involved in GR-induced suppression of CRH expression is through direct binding to a nGRE in the promoter region of CRH gene and subsequent recruitment of repressor complexes. In a rat hypothalamic cell line, it was demonstrated that Dex-induced CRH repression occurs through coordinated actions of corepressors involving Methyl-CpG-binding protein 2 (MeCP2), HDAC1, and DNA methyltransferase 3B (DNMT3B). Possibly, GR bound to nGRE recruits DNMT3B to the promoter in order to methylate a specific region, subsequently binding MeCP2 on these methylated sites followed by the recruitment of chromatin modify corepressor HDAC1, ultimately resulting in CRH suppression. Another possibility is that 2 independent complexes, one consisting of GR with DNMT3 for the methylation and the other the MeCP2, bound to methylated region, interact with HDAC1 to induce repression (68).

### Clock Genes

The clock system and the HPA axis are interconnected regulatory systems. Cortisol circadian rhythm is modulated by the interaction between a central pacemaker, located in the hypothalamic suprachiasmatic nuclei, and the HPA axis (69). At the molecular level, mediators of the clock system and cortisol also communicate with each other, both acting as transcription factors of many genes to influence cellular functions.

In CS, the impact of chronic GC exposure on clock genes expression was recently evaluated using peripheral blood samples from patients with active disease compared with healthy subjects. The circadian rhythm of peripheral clock gene expression (CLOCK, BMAL, PER1-3, and CRY1) was abolished as a result of hypercortisolism, and that may contribute to metabolic disorders observed in Cushing patients (70). Another study, which investigated persistent changes induced by hypercortisolism in visceral adipose tissue, found that the expression of clock genes, such as *PER1,* remained altered in association with persistent epigenetic changes in both H3K4me3 and H3K27ac induced by hypercortisolism even after the resolution of hypercortisolism (12). This suggests that chronic exposure to GC may induce sustained epigenetic changes that can influence clock genes expression. Nevertheless, further studies are warranted to better elucidate how long-term exposure to GC impacts clock genes expression using the epigenetic machinery.

## Glucocorticoid Effects on MicroRNAs

Along with histone modification and DNA methylation, microRNAs (miRNAs) have emerged as an epigenetic mechanism capable of impacting gene expression without changing DNA sequence (15). Interestingly, miRNA expression itself is also under the influence of epigenetic modifications through promoter methylation like any other protein-encoding genes (71).

MicroRNAs are small (about 20-25 nucleotides in length) non-coding RNAs that are important in transcriptional silencing of messenger RNA (mRNA). By partially pairing with mRNA, miRNAs can either induce mRNA degradation or inhibit mRNA translation to protein. MiRNAs regulate the translation of about 50% of the transcriptome, allowing them to play an important role in a wide range of biological functions, such as cell differentiation, proliferation, metabolism, and apoptosis under normal physiological and pathological situations. Some miRNAs can be classified as oncogenes or tumor suppressing genes, and aberrant expression of miRNAs may be implicated in tumor pathogenesis (71-73).

Insight into the regulation of miRNA expression is, therefore, crucial for a better understanding of tumor development and other human diseases, including cardiac, metabolic, and neurological disorders (73, 74). There are different regulatory mechanisms involved in miRNA expression, including transcriptional factors such as GR-GC. GC may modulate miRNA expression through direct binding to GRE in the promoter region of the host gene, as observed in hemopoietic tumor cells (75). In addition to transcriptional activation, in vascular smooth muscle cells, Dex treatment induces downregulation of DNMT1 and DNMT3a protein levels and reduces the methylation of miRNA-29c promoter, resulting in an increased expression of miRNA-29c (76). Interestingly, it was demonstrated that the increased expression of miRNA-29 family (miRNA-29a, -29b, and -29c) associates with metabolic dysfunction, such as obesity and insulin resistance, which pertains to CS (77, 78). With regard to metabolic dysfunction, miRNA-379 expression was shown to be upregulated by GC and its overexpression in the liver resulted in elevated levels of serum triglycerides associated with very low-density lipoprotein (VLDL) fraction in mice (79). In obese patients, the level of hepatic miRNA-379 expression was higher compared to nonobese patients and positively correlated with serum cortisol and triglycerides (79). Hence, GC-responsive miRNA may be, at least in part, a mediator to GC-driven metabolic conditions in CS.

In pathological conditions, such as seen in CS, prolonged exposure to an elevated cortisol level results in a wide range of comorbidities. It can be hypothesized that the chronic and excessive glucocorticoid levels may induce an aberrant miRNA expression that might impact several cellular processes related to bone and cardiometabolic disorders. A recent study addressed the impact of hypercortisolism on bone miRNA of patients with active CD compared to patients with nonfunctional pituitary adenomas. Significant changes in bone miRNA expression levels were observed, suggesting that the disruption of miRNA may be partially responsible for reduced bone formation and osteoblastogenesis (80). Similarly, altered expression levels of selected miRNAs related to endothelial biology in patients with CS may point to a contribution to a high incidence of cardiovascular disorders in Cushing patients (81). Therefore, dysregulated miRNAs as a consequence of high cortisol levels may underpin the development and progression of comorbidities related to CS. To the best of our knowledge, it is currently not clear whether miRNA dysregulation persists after resolution of hypercortisolism, thus contributing to the persistence of some comorbidities. This hypothesis needs to be further investigated.

MicroRNA can also be used as a diagnostic tool in CS. A study was performed to identify circulating miRNA as a biomarker to differentiate patients with CS from patients with suspected CS who had failed diagnostic tests (the control group) (82). It was observed that miRNA182-5p was differentially expressed in the CS cohort compared to the control group; therefore, it may be used as a biomarker (82). However, a large cohort is necessary to validate this finding (82). In corticotroph tumors, downregulation of miRNA 16-1 expression was observed relative to normal pituitary tissue (83). In contrast, the plasma level of miRNA16-5p was found to be significantly higher in CD compared to ectopic Cushing (EAS) and healthy controls (84). This finding suggests that miRNA16-5p may be a biomarker capable to differentiate the 2 forms of ACTH-dependent Cushing (84).

## Epidrugs and Glucocorticoid Action in Cushing's Syndrome

The interest in understanding the epigenetic mechanism of GC action in the context of CS is based on reversibility of epi-marks, such as DNA methylation and histone modifications, using epidrugs (85, 86). The biological characteristics of epigenetic drugs and their target have been extensively explored. Their effectiveness as antitumor drugs have been tested on corticotroph tumors using in vitro studies (87-89). However, a limited number of studies have explored the role of epidrugs as a therapeutic tool in reversing the genomic action of GC in CS, particularly in comorbidities induced by hypercortisolism (90, 91).

The use of histone deacetylase inhibitors (HDACi) may reduce the genomic action of GC (90-92). It has been demonstrated that the use of the HDAC inhibitor valproic acid increases the acetylation level of GR, consequently attenuating the genomic action of GC. In an experimental Cushing model in rats, the use of valproic acid decreased expression of genes related to lipogenesis, gluconeogenesis, and ion regulators in the kidney that ultimately reduces hepatic steatosis, hyperglycemia, and hypertension in ACTH-infused rats (90, 91).

More studies evaluating the effects of epidrugs influencing the GC actions are warranted to further elucidate the underlying mechanisms and to explore potential treatment modalities to reverse long-lasting consequences of chronic corticoid exposure.

## Conclusions

In physiologic conditions, GC are secreted in pulses following a circadian rhythm pattern, as opposed to a constant, chronic, and high GC exposure in CS. This pathological pattern may account for numerous devastating effects observed in CS (7). Yet, the expressed genome in response to chronic GC exposure may potentially be abnormal, leading to dysregulation in clock genes, among other effects.

GC levels may return to a normal circadian pattern in response to a successful treatment, but with incomplete reversibility of some CS features, which may in part be explained by epigenetic changes. The epigenetic machinery is used by GC to induce dynamic changes in chromatin to modulate gene expression. (Fig. 2) It seems that most of chromatin modifications are reversible, but some may persist resulting in long-term epigenetic changes. (Table 1)

**Table 1.** Evidence of interaction between glucocorticoid and epigenetic machinery

Epigenetic changes/epigenetic enzymes	Action
Histone acetylation (HAT)	Glucocorticoid receptors (GR) recruit co-regulators, such as CREB binding protein (CBP), which has intrinsic histone acetyltransferase (HAT) activity that modifies the chromatin structure from an inactive to an active state (25, 33, 35).
Histone deacetylation (HDAC)	GR recruit histone deacetylases (HDACs) to turn chromatin less accessible and suppress gene transcription (25, 35). The trans-repression process on POMC transcription achieved by glucocorticoids (GC) involves the histone deacetylation enzyme (HDAC2). GC mediates the upregulation of HDAC2 in rats exposed to chronic stress (40).
Histone demethylase (JMJD3)	GC suppress transcription of JMJD3 in endothelial cells treated with TNFα (41).
Histone modifications	Using ChIP-seq, a study in mice treated for 5 weeks with corticosterone showed higher levels of histone modifications (H3K4me3, H3K27ac) compared to control mice. In mice after a 10-week washout period, persistence of this epigenetic fingerprint was observed, which was associated with long-lasting changes in gene expression (12).
DNA methylation (DNMT3B) and histone deacetylation (HDAC1)	GC mediates CRH downregulation through DNMT3B to the promoter in order to methylate a specific region and recruitment of chromatin modify corepressor HDAC (68).
DNA hypomethylation	GC induces downregulation of DNMT1 in AtT20 (mouse corticotroph adenoma cell line) (20). GC induces upregulation of TET enzyme expression which was described in retinal and osteocyte cell line model (42, 43). An experimental study in mice previously exposed to high levels of GC showed differentially methylated regions (DMR) induced by GC treatment, of which the majority was loss of the methylation (11). Reduced DNA methylation in *FKBP5* gene was found in patients in active disease and also in remission state of Cushing syndrome (CS) as compared to a healthy control group (10). A genome-wide DNA methylation analysis showed a lower average of DNA methylation in patients in remission of CS compared to controls (9). A study using whole blood methylation profile demonstrated an association between cortisol excess and DNA hypomethylation in patients with CS (46).

Further studies are needed to elucidate how chronic exposure to GC leads to incomplete reversibility of CS morbidities via sustained modulation of the epigenetic machinery and possibly other mechanisms. Subsequent identification of therapeutic targets may offer new perspective for treatments, for example, with epidrugs, aiming to reverse hypercortisolism-related comorbidities.

## Data Availability

Data sharing is not applicable to this article, as no datasets were generated or analyzed during the current study.

## Abbreviations

ACTH: adrenocorticotropic hormone
CD: Cushing disease
CRH: corticotropin-releasing hormone
CS: Cushing syndrome
DBD: DNA binding domain
Dex: dexamethasone
DMR: differential methylation region
DNMT: DNA methyltransferase
GC: glucocorticoid
GR: glucocorticoid receptor
GRE: glucocorticoid responsive element
HAT: histone acetyltransferase
HDAC: histone deacetylase
HPA: hypothalamic-pituitary-adrenal
LBD: ligand binding domain
miRNA: microRNA
NTB: N-terminal domain
POMC: pro-opiomelanocortin
